# Spectra–Stability Relationships in Organic Electron Acceptors: Excited-State Analysis

**DOI:** 10.3390/molecules30224392

**Published:** 2025-11-13

**Authors:** Yezi Yang, Xuesong Zhai, Yang Jiang, Jinshan Wang, Chuang Yao

**Affiliations:** 1 Key Laboratory of Extraordinary Bond Engineering and Advance Materials Technology (EBEAM) of Chongqing, School of Materials Science and Engineering, Yangtze Normal University, Chongqing 408100, China; 2 School of Materials Science and Engineering, Yancheng Institute of Technology, Yancheng 224051, China

**Keywords:** organic electron acceptors, spectra analysis, excited state, stability

## Abstract

The operational stability of organic solar cells critically depends on the excited-state characteristics of electron acceptor materials. Through systematic quantum chemical calculations on four representative acceptors (PCBM, ITIC, Y6, and TBT-26), this study reveals fundamental spectra–stability relationships. Non-fullerene acceptors demonstrate superior light-harvesting with systematically tuned energy levels and significantly lower exciton binding energies (2.05–2.12 eV) compared to PCBM (2.97 eV), facilitating efficient charge separation. Structural dynamics analysis uncovers distinct stability mechanisms: ITIC maintains exceptional structural integrity (anionic RMSD = 0.023, S_1_ RMSD = 0.134) with superior bond preservation, ensuring balanced performance–stability. Y6 exhibits substantial structural relaxation in excited states (S_1_ RMSD = 0.307, T_1_ RMSD = 0.262) despite its low exciton binding energy, indicating significant non-radiative losses. TBT-26 employs selective bond stabilization, preserving acceptor–proximal bonding despite considerable anionic flexibility. These findings establish that optimal molecular design requires both favorable electronic properties and structural preservation in photoactive states, providing crucial guidance for developing efficient and stable organic photovoltaics.

## 1. Introduction

Organic electron acceptors play a pivotal role in the advancement of organic solar cells (OSCs) and other optoelectronic devices. OSCs have attracted significant attention over the past few decades due to their potential for low-cost, lightweight, flexible, and large-area photovoltaic applications [1,2]. A critical component dictating the performance and long-term stability of OSCs is the electron acceptor material [3]. Historically, fullerene derivatives, particularly phenyl-C61-butyric acid methyl ester (PCBM) and its analogues, have served as the benchmark electron acceptors in bulk heterojunction (BHJ) OSCs [4,5]. However, fullerenes suffer from limitations such as limited tunability of energy levels, weak absorption in the visible and near-infrared regions, and inadequate morphological stability, which collectively hinder further efficiency enhancements and device longevity [3]. The power conversion efficiency (PCE) of fullerene-based OSCs showed a slower pace of increase compared to non-fullerene counterparts over the years.

In recent years, non-fullerene acceptors (NFAs) have revolutionized OSCs research, enabling PCE exceeding 19% [6,7]. This remarkable progress is largely attributed to the rational molecular design of NFAs, which allows for precise tuning of their electronic structure, absorption profiles, and molecular packing characteristics [8,9,10]. Key design principles for NFAs include an acceptor–donor–acceptor (A-D-A) architecture, often featuring strong electron-donating central units and weak electron-withdrawing end groups to modulate molecular symmetry and conjugation length [11,12,13]. Among the prominent NFA classes, ITIC (3,9-bis(2-methylene-(3-(1,1-dicyanomethylene)indanone))-5,5,11,11-tetrakis(4hexylphenyl)dithieno[2,3-d:2′,3′-d′]-s-indaceno[1,2-b:5,6-b′]dithiophene) and its derivatives have gained significant attention [14,15]. ITIC, characterized by its fused-ring core and strong electron-withdrawing end groups, offers broad absorption in the visible and near-infrared regions and tunable energy levels. A more recent breakthrough in NFA design is Y6 [16], which has established new records for OSC efficiency [17]. Y6 features a unique fused-ring ladder-type core with strong electron-withdrawing terminal groups, leading to a highly planar and ordered molecular structure [18]. This structural characteristic contributes to efficient charge transport and superior light-harvesting capabilities, extending into the near-infrared region [19]. The successful implementation of Y6-based devices underscores the importance of carefully engineered molecular structures for optimizing photovoltaic performance.

The pursuit of low-cost, high-performance NFAs is crucial for the industrialization of OSCs. While fused-ring NFAs have led to remarkable PCEs exceeding 19%, their complex multi-step synthesis significantly increases material costs, hindering large-scale production. In contrast, non-fused NFAs, particularly those with a thiophene–benzene–thiophene (TBT) central unit, offer a promising low-cost alternative due to their simple synthetic routes. Yang et al. developed a completely non-fused acceptor named TBT-26, which features a para-alkoxy-substituted benzene unit and 1st-position branched alkoxy side chains [20]. As a result, TBT-26-based OSCs achieve an outstanding PCE of 17.0%—the highest reported for completely non-fused acceptors—along with a high fill factor of 0.807 and a remarkable short-circuit current density of 26.1 mA cm^−2^. Combined with a low estimated material cost of ~12 $/g and superior photostability, TBT-26 represents a major step toward cost-effective and efficient OPV technologies.

The continuous development of novel materials has significantly enhanced the PCE of OSCs while reducing fabrication costs. However, stability limitations now constitute the primary barrier to commercial deployment of organic photovoltaics. Crucially, recent experimental studies have established that the photostability of NFAs is governed by distinct yet interconnected molecular-level mechanisms. Luke et al. revealed that photoinduced conformational twisting around the core–acceptor dihedral acts as a critical initial step in the degradation cascade [21]. Speller et al. demonstrated that a shallow LUMO energy level facilitates superoxide formation via electron transfer to environmental oxygen, leading to rapid photo-oxidation of the blend [22]. Guo et al. systematically quantified the photobleaching rates of numerous NFAs, finding that most degrade faster than fullerenes, and highlighted the challenge of mitigating this through material design alone [23]. These findings collectively underscore that efficiency and operational stability are intrinsically linked to the electronic structures and excited-state dynamics of electron acceptors. To address stability bottlenecks, rational material design necessitates a fundamental understanding of multiscale structure-property relationships, specifically how molecular configurations govern electronic behavior and subsequent photophysical processes. In this context, quantum chemical calculations provide indispensable insights by simulating electronic structures and predicting spectroscopic properties and degradation pathways of organic semiconductors [24,25,26].

This study aims to delve into the intricate spectra–stability relationships in organic electron acceptors. The findings establish that optimal stability requires not only favorable electronic properties but also structural preservation in both photoactive and charged states, providing crucial design principles for developing next-generation organic electron acceptors that balance high efficiency with long-term operational stability.

## 2. Results and Discussion

### 2.1. Molecular Structures and Frontier Molecular Orbital Analysis

**Figure 1:**
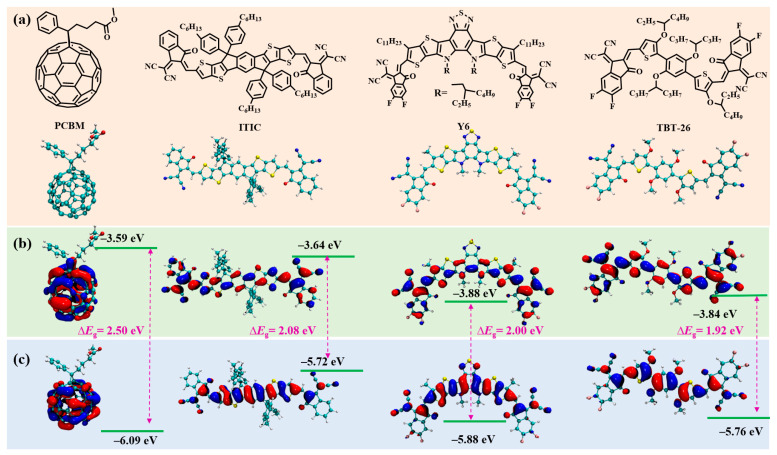
(**a**) Chemical structures and ground-state optimized geometries; (**b**) HOMO and (**c**) LUMO energy levels and orbital isosurfaces (0.02 a.u.) for PCBM, ITIC, Y6, and TBT-26.

To elucidate the fundamental electronic properties governing the performance and stability of organic electron acceptors, a comparative analysis of the ground-state optimized geometries and frontier molecular orbitals (FMOs) of PCBM, ITIC, Y6, and TBT-26 was conducted. The chemical structures and spatial distributions of the highest occupied molecular orbitals (HOMOs) and the lowest unoccupied molecular orbitals (LUMOs) are presented in Figure 1, providing critical insights into their electronic characteristics and their implications for device operation and stability.

The calculated HOMO and LUMO energy levels reveal distinct electronic characteristics among the four acceptors. The fullerene derivative PCBM exhibits the deepest-lying HOMO level at −6.09 eV and a LUMO at −3.59 eV, resulting in a relatively large electronic bandgap (*E*_g_) of 2.50 eV, which are in agreement with the reported results (HOMO: −6.1 eV; LUMO: −3.7 eV) [27]. This large bandgap is consistent with PCBM’s known weak absorption in the visible region [28], a characteristic that often necessitates complementary light-harvesting strategies in fullerene-based OSCs.

In contrast, NFAs demonstrate a systematic modulation of their energy levels, which is crucial for achieving high PCEs in OSCs [29]. ITIC, an early prominent NFA, possesses a HOMO/LUMO level of −5.72/−3.44 eV (*E*_g_ = 2.08 eV). The state-of-the-art fused-ring acceptor Y6, widely recognized for pushing OSC efficiencies to unprecedented levels, exhibits a slightly lower-lying HOMO at −5.88 eV and a LUMO at −3.88 eV, yielding a bandgap of 2.00 eV. Notably, the completely non-fused acceptor TBT-26 exhibits a moderate HOMO energy level at −5.76 eV and a LUMO level of −3.84 eV. This configuration results in the smallest computed bandgap of 1.92 eV within this series of electron acceptors. These results are consistent with reported trends (Table S1), e.g., NFAs show depper LUMO and higher HOMO than PCBM, supporting their red shift of absorption spectral.

A deeper HOMO level is generally associated with enhanced oxidative stability, a critical factor for the long-term performance of OSCs [30]. This characteristic could be a crucial determinant in the improved photostability reported for Y6 and PCBM, as deeper HOMO levels make the molecule less susceptible to oxidation by atmospheric oxygen or other reactive species. The significantly larger bandgap of PCBM compared to ITIC, Y6, and TBT-26 suggests a distinct strategy in photon management. This may involve a trade-off where the larger bandgap, while limiting light absorption in certain regions, could lead to a higher open-circuit voltage (*V*_oc_) and enhanced stability, albeit potentially at the expense of maximum current generation. This observed trend aligns well with experimental findings regarding the performance and stability of these classes of materials in photovoltaic devices [31]. Such systematic tuning of FMO through chemical structural engineering is a key approach to designing efficient small molecule acceptors for OSCs, as it allows for precise control over electronic properties and material calibration.

### 2.2. Excited-State Properties and Charge-Transfer Characteristics

**Figure 2:**
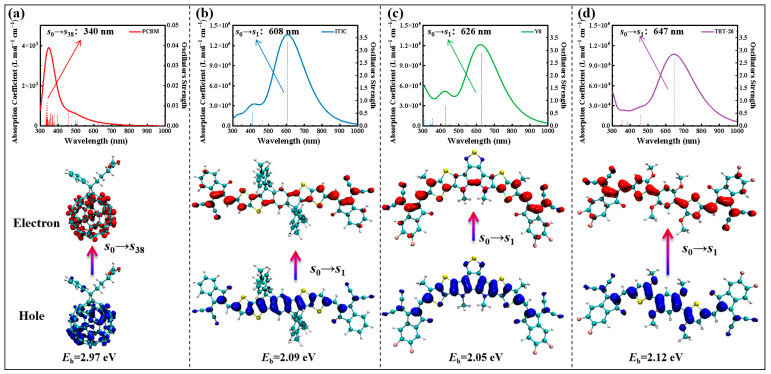
TD-DFT-calculated excited states contributing to absorption spectra of (**a**) PCBM, (**b**) ITIC, (**c**) Y6, and (**d**) TBT-26. Electron (red)/hole (blue) distributions for dominant transitions (highest oscillator strength) are shown below.

To gain deeper insight into the photophysical behavior and charge separation capabilities of the acceptors, we employed time-dependent density functional theory (TD-DFT) to simulate the key excited states contributing to their absorption spectra. Figure 2 presents the calculated spectra alongside the electron–hole distributions for the dominant electronic transition (i.e., the transition with the highest oscillator strength) for each molecule.

The simulated absorption spectra reveal distinct features that not only align with but also expand upon the insights derived from the ground-state FMO analysis. PCBM exhibits a high-energy transition at 340 nm (*S*_0_ → *S*_38_), which is characteristic of its localized π-π* transitions within the fullerene cage. This observation is consistent with its weak absorption in the visible light region, highlighting a fundamental difference in light-harvesting mechanisms compared to NFAs. While photochemical processes typically proceed from the lowest excited state (*S*_1_ or *T*_1_) according to Kasha’s rule, the initial photoexcitation in PCBM occurs at higher states due to its localized electronic transitions. The discussion of higher excited states here is relevant for understanding the absorption characteristics and initial charge generation. In stark contrast, the NFAs (ITIC, Y6, and TBT-26) all display dominant transitions in the visible to near-infrared region (NIR), which are predominantly identified as the *S*_0_ → *S*_1_ transition. The absorption maxima for these NFAs follow the trend of their optical bandgaps, with TBT-26 and Y6 absorbing at the longest wavelengths (647 nm and 626 nm, respectively), followed by ITIC (608 nm). It is important to note that these gas-phase TD-DFT calculations systematically blue-shift the absorption maxima compared to experimental values recorded in solid-state films (e.g., ~760 nm for TBT-26 [20], ~830 nm for Y6 [32], and ~700 nm for ITIC [32]). This discrepancy is primarily attributed to the absence of aggregation and dielectric screening effects in this gas-phase model, which are prominent in the condensed phase and lead to significant spectral red-shifts. This extended absorption into the visible region and NIR is a critical advantage of NFAs, enabling more efficient light harvesting across the solar spectrum, a key factor in achieving high PCEs in modern OSCs [13,20,33].

A critical parameter derived from these calculations is the electron–hole Coulomb attraction energy, which serves as a computational descriptor for the intrinsic exciton binding energy (*E*_b_) in the gas phase. This quantity represents the unscreened Coulombic interaction between the photogenerated electron and hole, and is calculated here using Multiwfn software 3.8.0 [34] for the dominant electron transition. It is important to note that this value corresponds to the binding energy in a vacuum, which is inherently larger than the effective *E*_b_ measured in solid-state films or calculated with environmental screening, where dielectric shielding significantly reduces the binding energy [35]. The results show a significant variation: PCBM has the highest *E*_b_ of 2.97 eV, a consequence of its highly localized, low-dielectric excitons. The NFAs exhibit substantially lower binding energies, with ITIC, Y6, and TBT-26 having values of 2.09 eV, 2.05 eV, and 2.12 eV, respectively. The lower *E*_b_ in NFAs is favorable for efficient exciton dissociation into free charges in a photovoltaic device. Notably, Y6 demonstrates the lowest *E*_b_ among the NFAs studied, which may contribute to the high fill factor observed in its corresponding OPV devices.

The electron–hole distribution maps for the dominant transition provide a direct visual representation of the initial charge separation upon photoexcitation. For the A-D-A type NFAs (ITIC, Y6, TBT-26), a clear charge-transfer (CT) character is observed. In both ITIC and Y6, the hole density (represented in blue) is primarily localized on the central donor core, while the electron density (represented in red) is delocalized over the entire molecule. This pronounced spatial separation between the electron and hole indicates the formation of a strong intramolecular CT state. Such well-defined CT states are crucial for facilitating efficient initial charge separation, a prerequisite for effective charge transport and collection in photovoltaic devices.

### 2.3. Structural Dynamics Analysis

**Figure 3:**
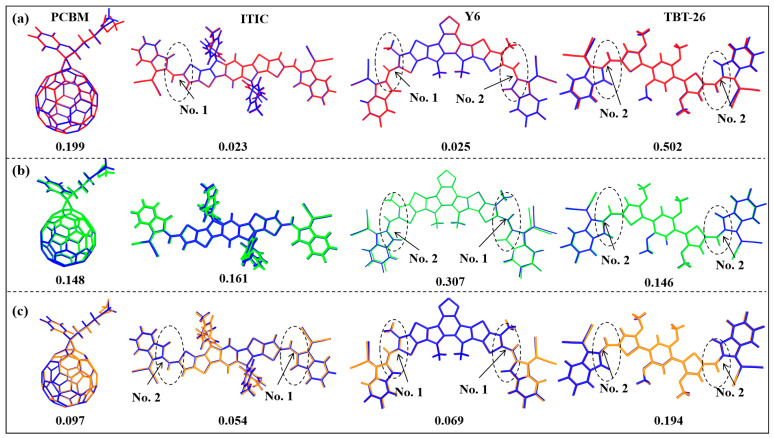
Structural differences between ground state (blue) and (**a**) anionic state (red), (**b**) first excited singlet state (*S*_1_, green) and (**c**) first excited triplet state (*T*_1_, orange). Corresponding RMSD values are quantified below.

To further elucidate the relationship between molecular structure, excited-state characteristics, and operational stability, we investigated the structural evolution from ground to excited and charged states. The extent of structural deformation in these active states is a key indicator of potential photodegradation pathways, as large geometric fluctuations can facilitate non-radiative decay and trigger bond cleavage Figure 3 presents the structural differences between the ground state (*S*_0_), anionic state, first excited singlet state (*S*_1_), and first triplet state (*T*_1_), with corresponding Root-mean-square deviation (RMSD) values quantifying the geometric changes. RMSD quantifies the average distance between atoms in two molecular structures after optimal alignment. It is calculated as:

$$RMSD=\sqrt{\frac{1}{N}\sum_{i=1}^{N}\delta_{i}^{2}}$$

where δ_i_ is the distance between corresponding atoms in the reference (ground state) and target (excited or charged state) structures. RMSD values in this work reflect the extent of structural reorganization upon electronic excitation or charging, with higher values indicating greater flexibility and potential instability.

The RMSD analysis reveals distinct molecular-specific responses to electronic excitation and charge accumulation. For the anionic state, PCBM exhibits moderate structural reorganization (RMSD = 0.199), consistent with its known instability under operational conditions [36]. The fused-ring NFAs ITIC and Y6 demonstrate exceptional structural integrity with minimal geometry changes (RMSD = 0.023 and 0.025, respectively), indicating remarkable resilience to electron uptake. This exceptional rigidity in the charged state is a critical factor underpinning the superior operational stability reported for ITIC-based devices [31]. In stark contrast, TBT-26 shows the most significant structural change in its anionic state (RMSD = 0.502), suggesting considerable backbone relaxation to accommodate the extra electron, which represents a potential vulnerability for long-term charge transport stability.

The excited state analysis reveals a contrasting pattern. In the *S*_1_ state, Y6 undergoes substantial geometry change (RMSD = 0.307), indicating significant structural relaxation that could contribute to non-radiative energy loss. This pronounced flexibility in the photoactive state is consistent with observations of higher non-radiative losses in Y6, which can impact photostability by providing channels for energy dissipation that may lead to molecular degradation. Crucially, TBT-26 demonstrates superior rigidity in the photoactive *S*_1_ state (RMSD = 0.146), the lowest among all NFAs. This suppressed geometric fluctuation in the optically active state directly correlates with reduced non-radiative decay channels, explaining its high photovoltaic performance. The triplet state (*T*_1_) analysis provides additional insights into potential degradation pathways. PCBM, ITIC, and Y6 show small triplet-state distortion (RMSD = 0.097, 0.054, and 0.069), indicting its relatively stable triplet characteristics. Notably, TBT-26 exhibits considerable *T*_1_ structural change (RMSD = 0.194), exceeding its *S*_1_ distortion level, suggesting larger relaxation pattern in *T*_1_ state. This value is intermediate between its *S*_1_ and anionic state values, indicating a balanced structural response across different excited states.

### 2.4. Bond Order Evolution

To complement the structural dynamics analysis and gain deeper insight into the electronic origins of molecular stability, we performed Laplacian bond order (LBO) analysis [37] for the key C-C bonds in the π-bridge of ITIC, Y6, and TBT-26 across different electronic states. The preservation of bond strength, particularly in photoactive and charged states, is directly linked to resistance against photo-induced scission and oxidative degradation. Table 1 presents the quantitative LBO values for bond No. 1 (proximal to donor core) and bond No. 2 (proximal to acceptor end group) in *S*_0_, anionic, *S*_1_, and *T*_1_ states.

The LBO evolution reveals systematic electronic redistribution patterns that underpin the observed structural dynamics. In the anionic state, all three NFAs exhibit a convergent trend: significant weakening of the acceptor-proximal No. 2 bond (LBO reduction of 0.11–0.12) accompanied by slight strengthening of the donor–proximal No. 1 bond (LBO increase of 0.04–0.06). This pattern reflects substantial electron density redistribution toward the acceptor units upon electron addition, consistent with their electron-withdrawing nature. The pronounced electronic reorganization in TBT-26’s anionic state correlates with its substantial structural relaxation observed in the RMSD analysis.

The excited state analysis reveals crucial differences in bond strength preservation that correlate with photostability. In the *S*_1_ state, ITIC demonstrates the most favorable bond order profile, with minimal reduction in its No. 2 bond (from 1.57 to 1.52) and moderate retention of No. 1 bond strength (1.35 to 1.31). This exceptional bond preservation aligns with ITIC’s superior rigidity in the photoactive *S*_1_ state observed in structural analysis, explaining its efficient charge generation with minimal non-radiative loss and correlating with its documented high photostability. In contrast, Y6 exhibits more pronounced bond weakening in both *S*_1_ and *T*_1_ states, particularly for the No. 2 bond (*S*_1_: 1.54 → 1.48; *T*_1_: 1.54 → 1.47). This substantial bond order reduction correlates with Y6’s significant structural relaxation in excited states and may contribute to its higher non-radiative energy loss and potentially lower resistance to photochemical degradation despite excellent photovoltaic performance.

Notably, TBT-26 shows a unique asymmetric behavior: while its No. 1 bond experiences the largest reduction among all molecules in the S_1_ state (1.34 → 1.27), its No. 2 bond maintains excellent strength (1.56 → 1.53), comparable to ITIC. This suggests that TBT-26’s molecular design allows the donor–proximal region to absorb most of the excitation-induced stress while preserving the critical acceptor–proximal bonding, providing a mechanism for balancing structural flexibility with electronic stability.

The anion and triplet state analysis further illuminates degradation pathways. All molecules show bond order convergence in the anion and *T*_1_ state, with reduced disparity between No. 1 and No. 2 bonds, indicating more uniform electron distribution that may influence degradation processes.

## 3. Materials and Methods

All quantum chemical calculations were carried out by the ORCA Revision 5.0.1 program [38]. Geometry optimization of ground-state, anion and cation of all materials investigated in this work was carried out at B3LYP/def2-SVP level with geometrical counterpoise correction to remove artificial overbinding effects from the basis set superposition error [39]. Frontier molecular orbital energy levels were calculated at B3LYP/def2-TZVP level. Since the PBE0 hybrid functional can estimate the reasonable absorption spectrum of most organic materials using time-dependent density functional theory (TD-DFT) [40], we used PBE0/def2-SVPD calculation level to investigate absorption spectra and excited states of all materials in the gas phase. The specific number of excited states calculated (e.g., the lowest 50 singlet and triplet states) to ensure coverage of the relevant optical transitions. In order to account for the major parts of dispersion force contribution to the energy, the atom-pairwise dispersion correction (D3ZERO) [41] was used during this calculation. During these quantum chemical calculations, the alkyl chains were omitted to reduce the calculation cost.

## 4. Conclusions

This comprehensive quantum chemical investigation reveals distinct spectra–stability relationships in organic electron acceptors, establishing that molecular stability is governed by the synergistic interplay of electronic structure, excited-state dynamics, and structural preservation. ITIC emerges as the most balanced acceptor, demonstrating exceptional structural integrity across electronic states (anionic RMSD = 0.023, S_1_ RMSD = 0.134) combined with superior bond preservation in excited states (No. 2 bond LBO: 1.57 → 1.52), explaining its excellent photostability. While Y6 achieves the lowest exciton binding energy (2.05 eV) beneficial for charge generation, its substantial structural relaxation in both *S*_1_ (RMSD = 0.307) and *T*_1_ (RMSD = 0.262) states and pronounced bond weakening (No. 2 bond LBO: 1.54 → 1.48) indicate higher non-radiative loss pathways. TBT-26 exhibits a unique stability mechanism through selective bond stabilization, maintaining strong acceptor-proximal bonding (No. 2 bond LBO: 1.56 → 1.53) despite significant anionic state flexibility (RMSD = 0.502). These findings provide fundamental design principles for next-generation acceptors, highlighting that optimal stability requires not only favorable electronic properties but also structural preservation in both photoactive and charged states.

## Figures and Tables

**Table 1:** Laplacian bond orders of the key C-C bonds in the π-bridge for molecules ITIC, Y6, and TBT-26. The table lists the bond orders for: (No. 1) the C-C bond proximal to the donor core, and (No. 2) the C-C bond proximal to the acceptor end group.

	ITIC		Y6		TBT-26	
	No. 1	No. 2	No. 1	No. 2	No. 1	No. 2
*S*_0_	1.35	1.57	1.37	1.54	1.34	1.56
Anion	1.40	1.46	1.41	1.43	1.40	1.44
*S*_1_	1.31	1.52	1.34	1.48	1.27	1.53
*T*_1_	1.42	1.44	1.39	1.47	1.38	1.48

## Data Availability

Data are contained within the article and Supplementary Materials.

## Supplementary Materials

The following supporting information can be downloaded at: https://www.mdpi.com/article/10.3390/molecules30224392/s1, Table S1: HOMO and LUMO energy levels for PCBM, ITIC, Y6, and TBT-26.
