# Supplementary material for: Spectra–Stability Relationships in Organic Electron Acceptors: Excited-State Analysis
Source: Molecules. 2025 Nov 13;30(22):4392. doi: 10.3390/molecules30224392 (PMC12655616; doi:10.3390/molecules30224392)
Supplement: Supplementary file 1 [file molecules-30-04392-s001.zip › molecules-3969937-supplementary.pdf]

Supporting Information

Spectra–Stability Relationships in Organic Electron Acceptors: Excited-State Analysis

Yezi Yang [1], Xuesong Zhai [2,*], Yang Jiang [2], Jinshan Wang [2,*] and Chuang Yao [1,*]

[1] Key Laboratory of Extraordinary Bond Engineering and Advance Materials Technology (EBEAM) of Chongqing, School of Materials Science and Engineering, Yangtze Normal University, Chongqing 408100, China
[2] School of Materials Science and Engineering, Yancheng Institute of Technology, Yancheng 224051, China
* Correspondence: zhaixuesong198579@163.com (X.Z.); wangjinshan@ycit.cn (J.W.); yaochuang@yznu.cn (C.Y.)

Table S1. HOMO and LUMO energy levels for PCBM, ITIC, Y6, and TBT-26.

	HOMO (eV)	LUMO (eV)	Reference
PCBM	-6.10	-3.70	[1]
ITIC	-5.54	-3.84	[2]
Y6	-5.65	-4.10	[3]
TBT-26	-5.54	-4.16	[4]

References

[1] G. Tchutchulashvili, K. P. Korona, W. Mech, S. Chusnutdinow, M. Sobanska, K. Klosek, Z. R. Zytkiewicz, and W. Sadowski, *J. Nano. Res.* **2020**, *22*, 84.

[2] Y. Yang, Z.-G. Zhang, H. Bin, S. Chen, L. Gao, L. Xue, C. Yang, and Y. Li, *J. Am. Chem. Soc.* **2016**, *138*, 15011-15018.

[3] J. Yuan, Y. Zhang, L. Zhou, G. Zhang, H.-L. Yip, T.-K. Lau, X. Lu, C. Zhu, H. Peng, P. A. Johnson, M. Leclerc, Y. Cao, J. Ulanski, Y. Li, and Y. Zou, *Joule* **2019**, *3*, 1140-1151.

[4] N. Yang, Y. Cui, Y. Xiao, Z. Chen, T. Zhang, Y. Yu, J. Ren, W. Wang, L. Ma, and J. Hou, *Angew Chem Int Ed Engl* **2024**, *63*, e202403753.
